# Defeating Crypsis: Detection and Learning of Camouflage Strategies

**DOI:** 10.1371/journal.pone.0073733

**Published:** 2013-09-10

**Authors:** Jolyon Troscianko, Alice E. Lown, Anna E. Hughes, Martin Stevens

**Affiliations:** 1 Centre for Ecology and Conservation, University of Exeter, Cornwall Campus, Penryn, United Kingdom; 2 Department of Physiology, Development, and Neuroscience, University of Cambridge, Cambridge, United Kingdom; University of Sussex, United Kingdom

## Abstract

Camouflage is perhaps the most widespread defence against predators in nature and an active area of interdisciplinary research. Recent work has aimed to understand what camouflage types exist (e.g. background matching, disruptive, and distractive patterns) and their effectiveness. However, work has almost exclusively focused on the efficacy of these strategies in preventing initial detection, despite the fact that predators often encounter the same prey phenotype repeatedly, affording them opportunities to learn to find those prey more effectively. The overall value of a camouflage strategy may, therefore, reflect both its ability to prevent detection by predators and resist predator learning. We conducted four experiments with humans searching for hidden targets of different camouflage types (disruptive, distractive, and background matching of various contrast levels) over a series of touch screen trials. As with previous work, disruptive coloration was the most successful method of concealment overall, especially with relatively high contrast patterns, whereas potentially distractive markings were either neutral or costly. However, high contrast patterns incurred faster decreases in detection times over trials compared to other stimuli. In addition, potentially distractive markings were sometimes learnt more slowly than background matching markings, despite being found more readily overall. Finally, learning effects were highly dependent upon the experimental paradigm, including the number of prey types seen and whether subjects encountered targets simultaneously or sequentially. Our results show that the survival advantage of camouflage strategies reflects both their ability to avoid initial detection (sensory mechanisms) and predator learning (perceptual mechanisms).

## Introduction

Camouflage is perhaps the most widespread anti-predator defence in animals (reviewed by [1], [2]). Many of the main types of camouflage were broadly outlined more than 100 years ago [3]–[5], and provide longstanding textbook examples of natural selection (e.g. [6]). Despite this, only recently has research sought to quantitatively distinguish the types of camouflage that exist, how they work, and the survival value that each type confers [1]. However, this work has almost exclusively focussed on the advantage different camouflage types confer in preventing detection by naïve observers, despite the fact that in nature many predators will successively encounter multiple prey types of the same or similar species.

Previous research has clearly demonstrated that attack rates of prey by predators are influenced by predator perceptual processes and the proportion of different prey types in the population. Predators are well known to form search images for more common prey phenotypes, based on transient changes in selective attention to specific prey features [7]–[10]. This can lead to predators attacking common forms disproportionately more than rare morphs (apostatic selection) and drive the evolution of prey polymorphisms and fluctuations in morph frequency [11], [12]. However, to date, no research effort that we are aware of has sought to explicitly determine whether specific prey phenotypes or camouflage types are more or less resistant to predator learning and search image formation, or whether the success of certain camouflage types is influenced by perceptual effects arising from predator experience and different search strategies. While some types of camouflage may be powerful in preventing initial detection, they may be learnt more readily than other prey types. Therefore, the overall benefit of a camouflage strategy may reflect the combined outcome of preventing both initial detection (its success in exploiting sensory processes) and predator learning (including search images; perceptual changes in attention towards specific prey features with experience). In a previous study, we found initial evidence that some prey markings may facilitate improved performance in detection of prey types over time by human observers [13]. However, we did not conduct a series of experiments specifically to test these ideas or compare several camouflage types or learning paradigms. Here, we conduct experiments to determine whether some camouflage types result in different rates of predator success over successive encounters, using humans as model ‘predators’.

Probably the main type of camouflage and the basis for most other forms of concealment is background matching, whereby an animal matches the general colour and pattern of the environment or substrate [1]. Background matching has been shown to decrease the probability of detection in a wide range of artificial and natural systems (reviewed by [14]). However, a limitation of this strategy is that it leaves the appearance of the body outline intact. Disruptive coloration, on the other hand, specifically works by breaking up the shape of the object’s outline by exploiting edge detection mechanisms in early visual processing [15], [16]. In recent years, experiments have shown that disruptive coloration provides a survival advantage over and above background matching alone, based on field experiments with wild predators, aviary studies, and experiments with computer games and humans searching for hidden targets (e.g. [17]–[21]). Generally, most experiments are consistent in finding that disruptive coloration is most effective when targets are of high contrast, but that the value of disruption decreases when pattern contrasts or intensities start to exceed those found in the background range [18], [21], [22].

Recently another potential type of camouflage has received attention, so-called ‘distractive’ markings. Here, high-contrast isolated markings that occur away from the body edge may draw or distract predator attention away from the body outline, leading to a failure to detect the object itself [1], [13], [23]. Three studies have tested this theory, which remains highly controversial. First, Stevens *et al*. [23] presented wild birds with artificial targets marked with a single potentially distractive marking and monitored predation levels. They found that targets with markings survived worse than unmarked background matching controls, especially with markings of high contrast. Second, an aviary study by Dimitrova *et al*. [24] trained birds to find hidden targets with different shapes and contrasts against either high or low contrast backgrounds. They showed that birds took longer to find targets with shapes of high contrast. Finally, Stevens *et al*. [13] conducted both field trials and experiments with humans searching for targets on touch screens. They found that potentially distractive markings were either neutral or costly in decreasing detection times/survival, especially high contrast markings. In addition, they found evidence that distractive markings would increase the rate of predator learning, since humans had quicker reductions in detection times towards distractive targets than background matching ones over successive trials. This finding raised the possibility that camouflage types might vary both in how well they avoid initial detection, and how effectively they resist predator learning. For example, prey with potentially distractive markings could provide reference points for subjects to search for, increasing learning from one trial to the next. Conversely, because disruptive coloration impedes detection of salient body edges, it may reduce the potential to learn information about shape or boundaries, limiting the ability of predators to attend to salient features like prey shape. However, the high contrast markings that improve the efficacy of disruption could provide cues for learning.

In addition to prey appearance, the nature of encounters may affect learning opportunities. For example, learning to increase detection will be more difficult when predators sequentially encounter a wide range of prey types at low rates, compared to a single prey type encountered frequently. Previous work has shown that search image formation occurs when predators see runs of the same prey type but not when they encounter several prey types at random (e.g. [25]). Therefore, the degree of learning may be highly dependent on the experimental paradigm adopted.

The use of humans to test general principles of camouflage under controlled settings has proven valuable, and provides good agreement with the findings of field studies using birds [13], [18], [26]. In this study, we conducted four experiments with humans searching for hidden targets on touch screens to test how effective different camouflage types (disruptive, distractive, and background matching) are in preventing detection. In addition, we tested each subject over a series of trials and compared rates of learning (as measured by reduction in detection times over trials) among treatments. Across these experiments we varied how many prey types each subject was presented with, and whether prey encounters were sequential or simultaneous. We predicted that, in line with past work, distractive markings would be easiest to detect overall and incur faster rates of learning compared to other camouflage types. Conversely, disruptive coloration would be the hardest to find, especially when of high contrast. We expected that if disruptive targets incur differences in learning that they would be learnt more slowly than background matching prey due to a lack of information about body shape. Finally, we predicted that the strongest learning effects would arise in experiment 2 when subjects encounter prey simultaneously, and that the weakest learning effects would arise in experiment 3 when subjects encounter single prey sequentially but of many different camouflage types.

## Materials and Methods

### Participants

Human participants (n = 420) were asked to detect and capture computer-generated ‘moth’ targets on a touchscreen display. Subjects gave their consent to take part in the study orally, and by clicking the “start” button on the touchscreen before the trial commenced and no personal or sensitive information was collected. The University of Cambridge's ethical research policies were adhered to and no ethical review was required. Subjects were free to terminate the trial at any point without explanation.

### Stimulus Generation

To make the targets, twenty background images of oak tree bark were taken under natural lighting conditions using a Nikon D90 in Madingley Woods, Cambridgeshire. These images were converted to an 8-bit greyscale image and equalised to fill the 8-bit dynamic range, and rescaled to 1280×1024 pixels (maintaining a 1∶1 aspect ratio). Targets were automatically generated from the background image they were displayed against using self-written code in ImageJ ([27], version 1.46), ensuring that all 16,400 prey used in our experiments were unique. First, a triangular section 100 pixels wide by 50 pixels high was selected from a random location in the background bark image. A probability template was then applied to this triangle that linearly altered the likelihood of patterns from the background image touching the edge of the target. The background-matching targets matched the thresholded pattern of the background except that the dark patterns could not touch the target edge, whereas for the disruptive targets, we stipulated that at least some markings must touch the target outline [17], [18]. Median smoothing with a diameter of 1.5 pixels was used to eliminate single pixels appearing in the patterns. The targets were thresholded so that the dark pattern made up 40±1% of their area. Low contrast prey had light and dark pattern grey levels equal to the 67 and 49 percentile grey levels of the background image, while high contrast prey used values of 77.5 and 17.5 respectively. These values result in an average of 50% grey when the 40% pattern coverage is introduced, resulting in uniform prey brightness between target types that scales with the overall brightness of the background image (for example, a darker background image would be shown with darker targets, but all treatments on that background would have the same overall brightness). Unmarked prey were set at the 50 percentile (median) grey value. Extreme contrast prey had black patterns against a white background, corresponding to the extreme range of the background image. Potentially distractive markings were added to the prey by repeatedly sampling the background image with a thresholding selection tool until a selection with an area of between 50 and 60 pixels was found that had maximal width and height dimensions not exceeding 20 pixels. This shape was then coloured (either green or white) and placed randomly inside the prey item such that it was not within 2 pixels of the edge of the prey.

### Experimental Design

**Figure 1:**
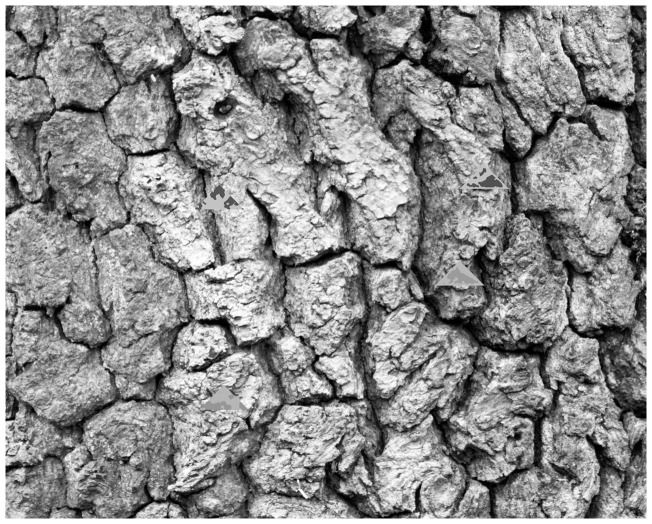
An example of the slide layout presented to participants in experiment 2, where participants were shown four treatments simultaneously. Target treatments (clockwise from upper left target): D-H, BM-H, BM-L and D-L. In all other experiments just one target was presented on each slide.

Each background image was presented to participants on a 1280×1024 pixel touchscreen display (Elo 1515L; Tyco Electronics, Shanghai, China for all experiments, except 4c which used an Iiyama Prolite T1931SR-B screen, Iiyama International, China) using applications generated in Multimedia Fusion (version 2). All images were displayed full-screen at full scale. Prey items were randomly positioned against the background they were generated from with the provision that they were not within 100 pixels of the display edge or 200 pixels of another prey item (in experiment 2), figure 1 shows an example slide. Participants were asked to capture the target as soon as they detected it by touching the screen. The software recorded the position of the prey and the participant’s touch, along with the timing of the capture to within 100^th^ of a second. If there was just one prey type per slide the application waited for one second and then progressed to the next slide. Where multiple prey were presented together in the same slide, each prey item disappeared once touched and progressed only once all prey were captured. If the participants failed to find the targets after a period of 15 seconds (or 30 seconds where multiple prey were presented on the same slide) they progressed to the next slide. The twenty different background images were presented in a crossed and balanced order such that for every 20 participants each background image was first once, and thereafter they were crossed to ensure an even presentation of backgrounds over the series of slides and no replication of slide ordering. Where participants were presented with different target types sequentially, the order of these treatments was randomised in six blocks of ten slides each. This was chosen to prevent learning of a fixed sequence order while maintaining uniform exposure to all treatments over the course of the trial.

### Experiment 1 – One-way between-subject

Each participant was shown one of nine different treatments during their trial, with one target per slide (n = 180 participants in total, 20 participants per treatment with 20 slides each). We used the following treatments: background matching low (BM-L), high (BM-H, and extreme (BM-E) contrast, disruptive low (D-L), high (D-H), and extreme (D-E) contrast, and background matching targets with either a single potentially distractive white (DI-W), or green (DI-G) marking. Finally, we had a uniform target (U). This experiment aimed to determine differences in overall detection times and learning over trials when subjects only ever encounter one target type.

### Experiment 2 – Four-way simultaneous

We presented each participant with four different treatment types simultaneously on the same slide (20 slides per subject, n = 40 participants). The treatments used were BM-L, BM-H, D-L, and D-H. Participants progressed to the next slide after all targets were found, or 30 seconds from first being shown the slide. Here, our aim was to test for differences in overall detection and learning across trials when subjects encounter multiple prey with different attributes at the same time. Figure 1 shows an example of the slide layout shown to participants.

### Experiment 3 – Five-way sequential

Participants encountered five different treatment types sequentially, with one treatment per slide (n = 80 participants with 60 slides each). We used BM-L, BM-H, D-L, D-H, and DI-W. The aim was to test whether there are differences in detection times across trials among treatments when subject were exposed to a range of prey types.

### Experiment 4 – Two-way sequential

Participants encountered two treatment types, presented sequentially with one target per slide (n = 40 participants with 60 slides each per version; n = 120 participants in total). For experiment 4a subjects were presented with BM-L and D-H, for 4b they received BM-L and DI-W, and for 4c they received BM-L and DI-G. We aimed to determine whether there are differences in learning over successive slides when subjects only encounter a small number of prey types sequentially.

### Statistical methods

Due to the repeated measures design of our experiment with random participants we modelled our data using generalised linear mixed models (GLMMs [28], [29]) in R ([30], version 2.15.1) using the LME4 package (version 0.999999-0), and p-values generated using MCMC implemented in LMERConvenienceFunctions (version 1.7). A log-normal error structure was specified throughout. Capture time was modelled against the interaction between treatment (each camouflage type and contrast level being a unique factor) and slide number (to test for differences in learning rates), and additional variables were included in each full model (as random effects where appropriate), these being: participant, background image, whether the participant was naïve regarding the general prey appearance (i.e. the first trial), and the minimum distance from the target to the edge of the screen. The models were then simplified based on AIC weights and log-likelihood to produce a best-fit model; starting with a full-interaction model, interactions and terms were removed in a stepwise fashion that improved the model fit [28], [29]. Treatment BM-L was taken as the baseline against which other strategies were compared, with further comparisons planned for the different contrast levels within the disruptive treatments.

## Results

### Experiment 1 – One-way between-subject

**Figure 2:**
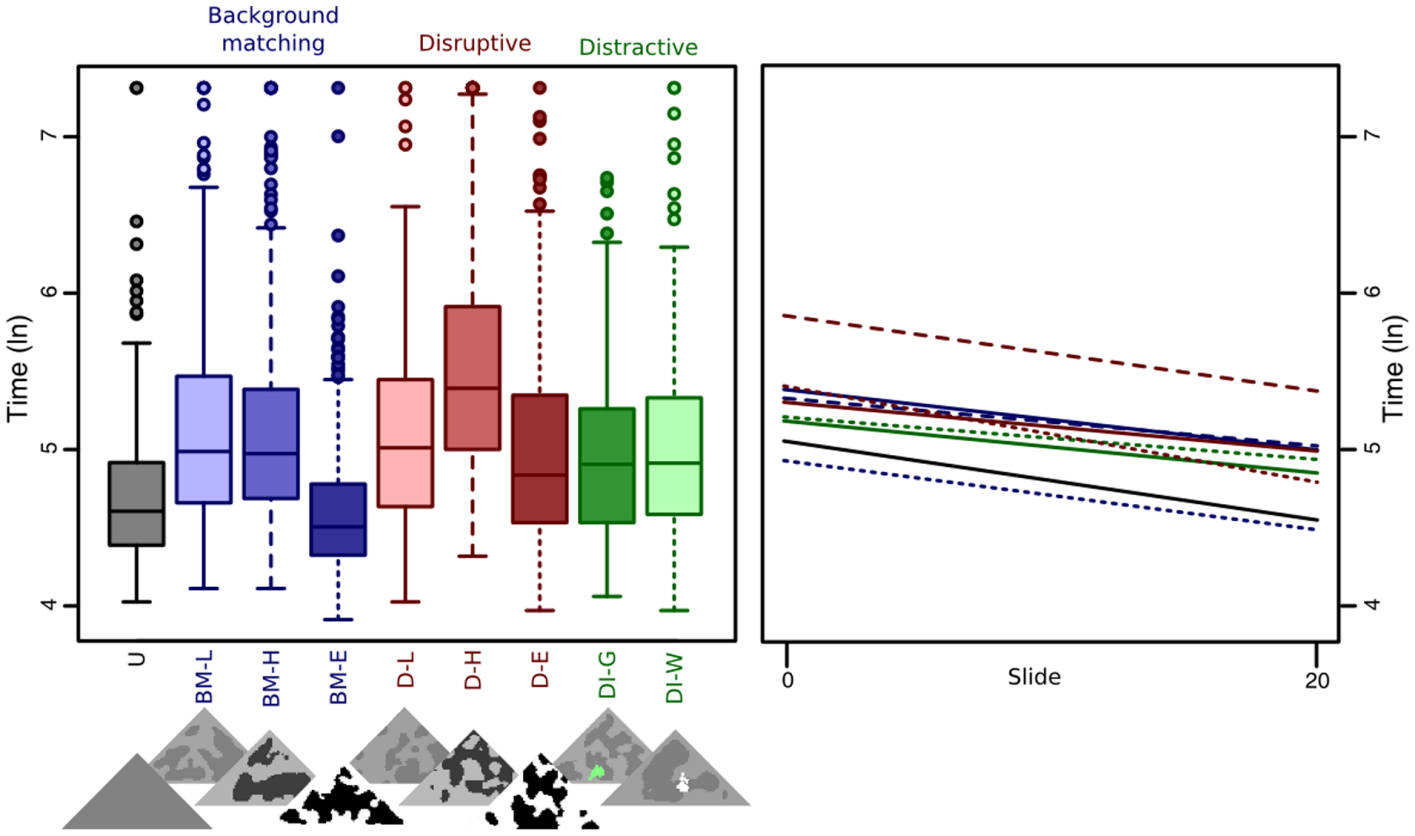
Capture times for treatments in experiment 1 across all slides (left, bars show quartile ranges), and regressions of capture times over the course of the trials (right). See main text for explanation of treatment codes. Logged capture times can be converted to 100ths of a second through taking the exponential.

There was no evidence for differential learning rates in experiment 1 (all p-values >0.05; figure 2), and this interaction was dropped from the model during simplification. The resulting best-fit model revealed differences in overall detection times compared to background matching low (BM-L): uniform (U) was captured significantly faster (t = −6.548, p<0.001), disruptive low (D-L) showed no significant difference (t = −0.485, p = 0.628), however, D-H took significantly longer to find (t = 6.720, p<0.001) and was the most difficult treatment to find of all. Increasing the contrast of the BM treatment from low to high resulted in no significant difference in detection times (t = 0.181, p = 0.857), however BM-E was captured significantly faster than BM-L (t = −7.949, p<0.001). Conversely the D-H was found significantly more slowly than D-L (t = 7.204, p<0.001). Although disruptive-extreme (D-E) was found faster than disruptive-low this effect was not significant (t = −1.866, p = 0.062). Distractive-white (DI-W) had detection times that were not significantly different to BM-L (t = −1.133 p = 0.258), but distractive-green (DI-G) was captured significantly faster than BM-L (t = −2.504, p = 0.012). In this experiment the most difficult targets to find from start to finish were the disruptive-high treatments.

### Experiment 2 – Four-way simultaneous

**Figure 3:**
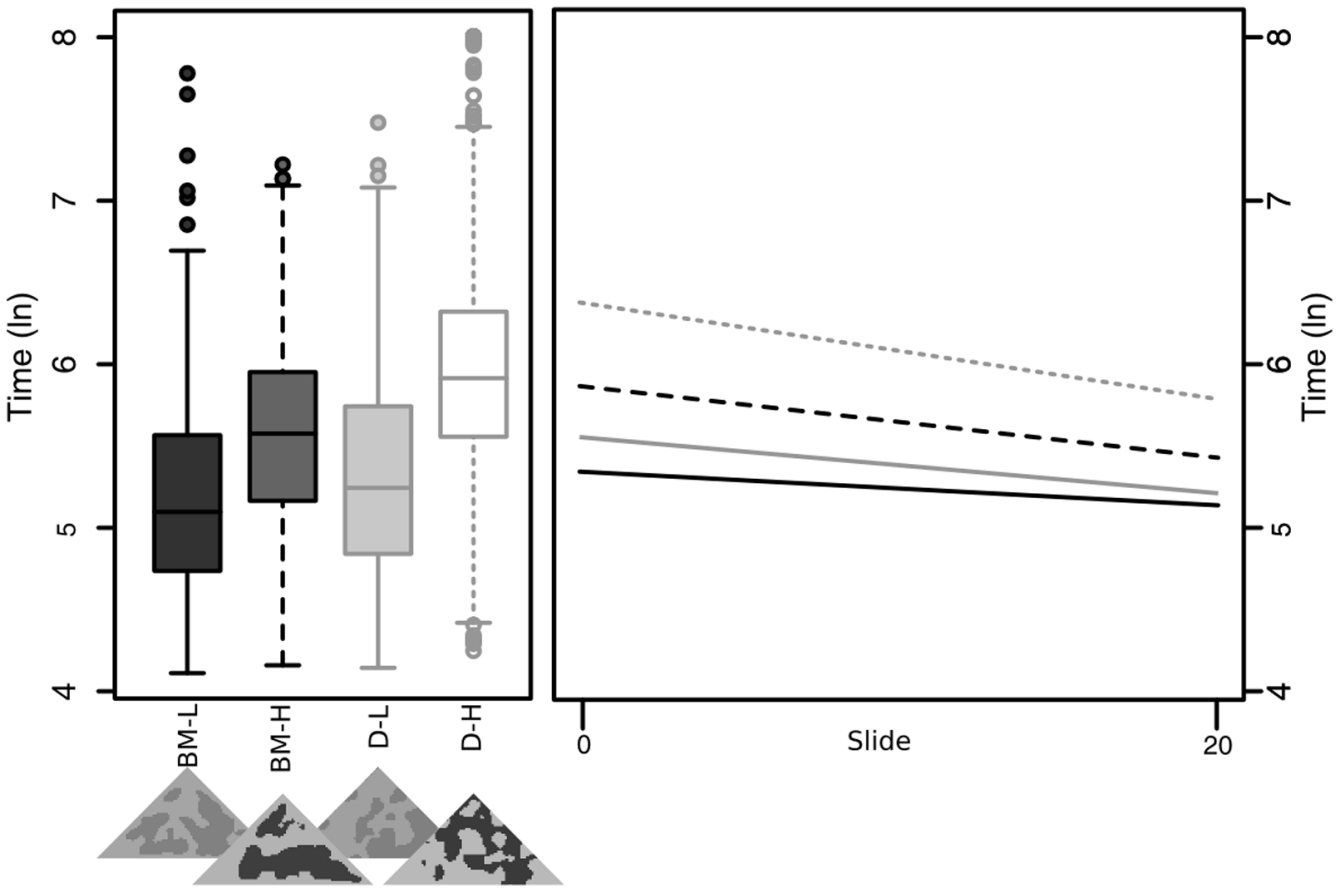
Capture times by treatment in experiment 2 across all slides (left, bars show quartile ranges), and regressions of capture times over the course of the trials (right).

There were significant differences in learning rates in the final best-fit model; participants learnt to capture both disruptive high (D-H) and background matching high (BM-H) faster than BM-L (t = −4.132, p<0.001; and t = −2.643, p = 0.008 respectively; figure 3), while there were no differences in learning rates between BM-L and D-L (t−1.635, p = 0.102). This suggests increased contrast makes it easier to learn to find prey (rather than a cost of the disruptive strategy itself). Over the course of this experiment D-H remained significantly more difficult to capture than BM-L (t = 13.315, p<0.001), and was the most successful strategy. BM-H and D-L both took, on average, longer to capture than BM-L (t = 7.484, p<0.001, and t = 3.699, p<0.001 respectively). As with experiment 1, disruptive-high remained the best strategy; however, the different learning rates suggest that background matching-low would eventually equalise, or even become a better strategy over successive encounters.

### Experiment 3 – Five-way sequential

**Figure 4:**
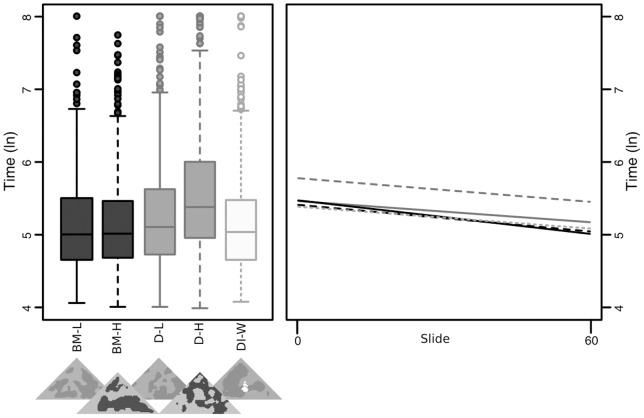
Capture times by treatment in experiment 3 across all slides (left, bars show quartile ranges), and regressions of capture times over the course of the trials (right).

We found no evidence for differential learning rates in experiment 3 (all p-values >0.05; figure 4), and the interaction was dropped from the model during simplification. The resulting best-fit model revealed overall differences between treatments; disruptive high (D-H) remained the most successful strategy, taking significantly longer to capture than background matching low (BM-L) (t = 14.913, p<0.001). D-L also took longer to find on average than BM-L (t = 4.167, p<0.001), however there was no significant difference in capture times between BM-L and BM-H or DI-W (t = −0.0488, p = 0.961, and t = 0.831, p = 0.406 respectively). D-H also took significantly longer to capture on average than D-L (t = 10.751, p<0.001).

### Experiment 4 – Two-way sequential

**Figure 5:**
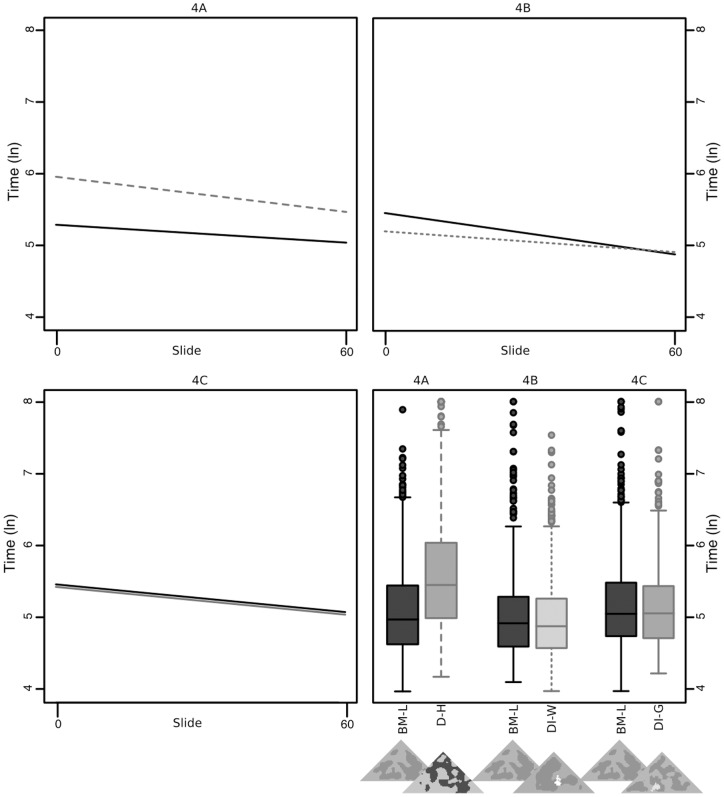
Regressions of capture times over the course of the trials (Experiment 4A: top left, 4B: top right, 4C bottom left), and overall capture times by treatment in experiments 4A, 4B and 4C, bars show quartile ranges.

In experiment 4a, we found marginal evidence for significantly higher rates of learning in disruptive high (D-H) than background matching low (BM-L) (t = −1.941, p = 0.052; figure 5). Overall capture times fell in line with previous findings, with D-H being found significantly more slowly than BM-L (t = 6.950, p<0.001). This finding falls in line with experiment 2, where disruptive-high was the best strategy throughout, but over successive encounters the background matching strategy could equalise with or even beat the disruptive strategy. In experiment 4b, participants learnt to capture BM-L significantly faster than distractive white (DI-W) (t = 2.328, p = 0.020; figure 5). However, DI-W was captured significantly faster than BM-L overall (t = −2.557, p = 0.011). Initially, the best strategy here was background matching, but after 60 trials the disruptive-white strategy had equalised, suggesting it would beat background matching over successive encounters. In experiment 4c there were no significant differences in learning rates or overall capture times between BM-L and DI-G (t = 0.642, p = 0.521, and t = −1.085, p = 0.278 respectively; figure 5).

Trial number was always an informative predictor of response times that was retained in all stepwise model simplifications, and was a highly significant predictor of reduced capture times over successive slides in all models where the interaction between trial number and treatment were dropped (in all cases p<0.001). The minimum distance from the target to the edge of the screen and participant naivety were also retained in all stepwise model simplifications, indicating they were highly informative predictors in our experiment. Therefore we would recommend the use of these variables in future touch screen search experiments.

## Discussion

Our experiments reveal that camouflage type influences the speed of overall detection, and also leads to differences in subject learning rates (measured as reduction in detection times over successive trials). The latter finding is influenced by the experimental paradigm, including the number of target types that subjects encounter and whether encounters of different target types are simultaneous or sequential.

For overall detection times, our results were in accordance with previous work. First, background matching decreased detection compared to an unmarked plain control (as with [17], [18], [21]). Second, targets with disruptive coloration took longer to find than other treatment types, and this was especially the case when the pattern was of high contrast. However, extreme levels of contrast led to decreased detection times for both disruption and background matching, in accordance with previous field studies and computer experiments [18], [21], [22]. Third, isolated ‘distractive-style’ markings of a conspicuous colour (green markings on achromatic prey) were costly or neutral compared to overall detection times in background matching targets. Stevens *et al*. [13] also found that targets with white distractive markings on generally green patterned prey were detected quickly. In experiment 4b targets had longer detection times when they had a white distractive marking.

Our most novel findings relate to subject learning. In both experiment 2, when prey types were presented simultaneously on the screen, and in experiment 4a when they were encountered sequentially, high contrast disruptive patterns were learnt more quickly than low contrast patterns. This is not a cost of disruption *per se*, but rather a cost of high contrast because in experiment 2 high contrast background matching targets also suffered greater reduction in detection times than low contrast equivalent targets. However, because disruption typically works best with high contrast [17], [18], [31], disruptive patterns may as a result be more prone to learning effects. As such, there may be a trade-off in having disruptive coloration, with high contrast patterns reducing initial detection, but facilitating predator learning if encounters occur frequently or increase. Where the balance lies will depend on the ecological circumstances. High contrast markings should be favoured when predators have limited opportunities to repeatedly encounter the same prey types, for example, in comparatively rare species with short-lived predators, in animals with many predator species, or where predators encounter many prey types. Conversely, high contrast markings would be costly when species face more specialist long-lived predators, or are highly abundant. Our results suggest that the widespread idea that high contrast is beneficial to disruption may be oversimplified when one considers interactions with predators over time. An important question for the future is at what point the benefit of high contrast to disruptive markings is lost.

In experiment 4b we found, unexpectedly, that prey with a single white marking had slower rates of subject learning than the background matching targets (although background matching targets still took longer to detect overall). The reason for this is unclear, but subjects may have adopted a strategy of searching for white markings and this was a good overall strategy as targets with white markings had shorter detection times. However, compared to simply searching for a triangular shape alone, subjects improved less in reducing detection times, perhaps because there were other bright isolated markings in the background that led to false detections. In contrast, when markings are genuinely conspicuous and beyond the background range then they have short detection times overall and are either easy to learn or there is no benefit to learning as they stand out clearly from the background already (see below). In accordance with this, we did not find any benefit in learning rates for the targets with green markings and in terms of overall detection they were either neutral or costly (as [13]). Alternatively, the addition of a white distractive marking to the target would increase the contrast of the target, and high contrast facilitated higher learning rates in experiments 2 and 4a. The green distractive markings would not have caused as large an increase in target contrast, perhaps explaining why we did not detect learning rate differences in experiment 4c. Furthermore, the combination of distractive markings and contrast levels in this achromatic experiment could explain why learning rates in distractive targets were not higher, in accordance with our previous study that used chromatic backgrounds [13].

Whether our findings relating to reduction in detection times should be referred to as ‘search images’ is debatable. Normally, a key component of search image theory is that in becoming better at finding one (relatively common) prey type a predator becomes worse at finding other prey types (e.g. [32], [33]). In our experiments, subjects always continued to improve at finding all prey types, even when the rate of improvement varied across treatments. Several studies have shown that search image effects are influenced by the degree of camouflage (e.g. [7]). Kono *et al*. [34] found that when detection performance was very good from the start then search images do not arise because there is little room for improvement. We would therefore expect to find search image formation more often with well camouflaged phenotypes, and our targets may not have achieved this level of difficulty. Furthermore, studies have often only found evidence of search image formation when predators face ‘runs’ of several of the same prey type in sequence before searching for other prey (e.g. [25]). In our experiments the subjects encountered equal proportions of prey types in a pseudorandom order. Without encountering long runs of the same treatment it might have been impossible for our subjects to form a search image. However, honing in on one prey type at the expense of others may not always be necessary, particularly when prey species share common defining features. Reid & Shettleworth [35] showed in pigeons searching for dyed wheat that an increase in experience in detecting one prey type also increased their ability to detect another prey type. They suggested that many search tasks are better understood by a mechanism of ‘attentional priming’, whereby subjects search for hidden targets among a series of distractors corresponding to elements in the background. If the target is distinctive, and differs from the distractors in at least one dimension (e.g. colour, pattern or brightness) then it will ‘pop out’ and the observer can rapidly search the whole visual field using a parallel search approach [36]. This is likely to explain our findings (as in [13]) that isolated conspicuous markings allow fast detection times as they immediately stand out from the background. However, when no single feature can be used to distinguish target from background, the task is more difficult and the observer must learn what features to use to discriminate prey from the background by forming associative links between them. Predators instead must use conjunction search, combining features (e.g. triangle shape and high contrast). By learning what features allow prey to be located most effectively, a predator can become better at locating other prey types too [35]. This type of conjunction search is more likely to be needed to find effectively camouflaged targets, such as those with disruptive or background matching patterns. Recent work also shows that when detection tasks are difficult, predators may use serial search approaches instead of rapid parallel search, and this can result in increased variation in prey phenotypes as a result of predator search image formation [12]. This could account for our findings; when the contrast was high our subjects would have been able to form links between the contrast cue and target shape, boosting learning rates, but when the contrast was closer to background levels this link could not be formed. Every prey type that each subject faced, even of the same treatment, had a unique pattern. Therefore, subjects cannot have been learning to find the specific markings found on any one treatment type but rather common features shared by them.

The experimental paradigm also affected differences in learning between treatments. We found strongest differences in learning when subjects encountered several prey types simultaneously (and in [13]). This indicates that subjects may adopt a search strategy to prioritise finding prey with more salient features first, resulting in those prey doing disproportionately badly over time. For example, subjects may adopt an approach searching for high contrast patches in the environment or discrete single markings. However, when subjects have uncertainty about what prey types they may encounter at any given time, and encounter many types (experiment 3), then it would seem logical to use a more general strategy such as ‘search for a hidden triangle shape’. In situations where the number of prey types encountered is low and more predictable, then specific search tactics for particular prey features may be possible again, as with experiment 4. Kono *et al*. [34] found that search image could be facilitated by associative cuing, whereby predators could learn to search for a specific prey type when faced with a particular background type. Our findings have implications for predator foraging because predators that encounter many prey types unpredictably in one patch type may not form search images for one prey type. Instead, they may search more generally for common features that can be used to find many types (such as overall shape). In contrast, when predators search in different niches or patches where different prey types are predictably found, they may be able to form search images for one prey type.

Here, we have shown that the overall value of different types of camouflage is affected not just by the relative value in preventing initial detection against naïve predators, but also by how readily predators improve in finding prey of specific phenotypes over multiple encounters. To date, work has almost entirely focussed on initial detection, yet given that in nature many predators will encounter many prey of the same types over time we feel that predator learning of prey phenotypes requires more research. Our experiments indicate that the overall value of a camouflage strategy is determined not just in terms of how it defeats predator sensory processing, but also how it defeats perceptual and cognitive processes. Thus, prey phenotypes that are good at defeating sensory processes (such as disruption and edge detection) may be poor at defeating cognitive processes. In future, work should investigate more comprehensively how specific features of prey can promote learning and search image formation, how this learning is affected by interactions between predators and prey, and how these combined effects might influence the evolution of prey camouflage strategies and appearance. In addition, it will be important to address the question of longer-term learning. Here, subjects undertook all the trials within a short space of time (typically less than 15 minutes). However, in nature, predators will often find have significant gaps between finding several prey of the same type (e.g. hours or days).
